# Effective and rapid treatment of wound botulism, a case report

**DOI:** 10.1186/s12893-017-0300-4

**Published:** 2017-10-26

**Authors:** M. Schulte, U. Hamsen, T. A. Schildhauer, T. Ramczykowski

**Affiliations:** Chirurgische Universitäts- und Poliklinik, BG-Klinikum Bergmannsheil GmbH, Universitätsklinikum der Ruhr-Universität, Bürkle-de-la-Camp-Platz 1, 44789 Bochum, Germany

**Keywords:** Wound botulism, *Botulinum*, Abscess

## Abstract

**Background:**

The latest news shows several cases of contaminated heroin that is found in different parts all over Europe. This information can be helpful for the emergency doctors to find the correct diagnosis of wound botulism in patients who are intravenous drug users.

**Case presentation:**

We describe a case of a 40-year-old man who presented to the emergency department in 2016. He suffered from mild dysarthria, diplopia, dysphagia and ptosis since two days. The CT-scan of the cerebrum and the liquor were without any pathological results. We found out that the patient is an intravenous drug user and the clinical examination showed an abscess in the left groin. So we treated him with the suspected diagnosis of wound botulism.

In the emergency operation we split the abscess, made a radical debridement and complementary treated him with a high dose of penicillin g and two units of botulism antitoxin. The suspected diagnosis was confirmed a few days later by finding the Toxin B in the abscess and in the patient’s serum. In the following days the neurological symptoms decreased and the wound healing was without any complications. The patient left the hospital after nine days; the antibiotic therapy with penicillin g was continued for several days. In a following examination, 14 days after the patient’s discharge of the hospital, no further symptoms were found and the abscess was treated successfully without any problems.

**Conclusion:**

Because wound botulism is a very rare disease it can be challenging to the attending physician. This case shows a fast treatment with full recovery of the patient without any further disabilities, which can be used for the future.

## Background

Wound botulism is a very rare disease. There are five different forms of acquisition with the toxin of *Clostridium botulinum*.

The most common form is the foodborne. It occurs from the ingestion of food containing preformed toxin.

The second one, the childhood botulism, comes from the production of toxin by germinating spores of *C. botulinum* in the infant’s intestine.

The enteric infectious botulism is diagnosed when there is no other acquisition of the toxin like food or due to contamination of a wound. It is supposed to occur especially after an antibiotic treatment, which detoriates the normal intestinal flora.

The fourth one is the inhaled botulism (aerosolized toxin) and the fifth one is the wound botulism. The last one is commonly represented in patients who are intravenous drug users (IDU) [1–3].

The Botulinum toxin first appeared in 1897. Today there are known seven immunologically different types of the toxin of *C. botulinum* (A-G), but only four are descripted in human diseases (A, B, E, F). For the wound botulism the serotype A and B are the most common ones. The toxins block the presynaptic acetylcholine receptor in the neuromuscular junction of the motor neuron. On the one hand it is used in different ways for medical or cosmetic treatments, on the other hand it is one of the most poisonous substances known [1].

There are no exact numbers of cases in a year for wound botulism in Europe, but there have been described different cluster in a short period of time and in a close local area. For example there were 12 cases in Rhineland, Germany, in the year 2005 and 25 about the late 2014 and the early 2015 in Scotland and Norway. All of these described cases were among people who had injected heroin. This heroin seems to be contaminated with the spores of *C. botulinum* [4–6].

## Case presentation

We describe a case report of a 40-year-old man who presented to the ED of a level-one-trauma center in 2016. He first was assigned to the neurological area on the base of suffering from several neurological symptoms. The patient was a normal weighting young man with a reduced general condition. The symptoms were present since two days. He had no additional chronic diseases except for intravenous drug use of heroin about ten years. The last use of heroin in his left groin was about ten days ago. The first examination showed a patient with normal conscious level. He felt “not well” and in the morning of the day he presented at the hospital he also noticed a dry mouth with a numb tongue.

He had a fever about 38,7 °C and suffered from several neurological symptoms, especially from cranial nerve dysfunction like ptosis, dysarthria and dysphagia. He had no paresis or paraesthesia in the upper or lower extremity and the Babinski sign was negative. Furthermore he had a normal blood pressure, no dyspnea or meningism. The clinical examination showed a typical abscess formation from about 10 × 10 cm in the left groin region and a tenderness of palpation of the inguinal lymph node. The first time the patient noticed this formation was ten days ago and he remarked that it has grown and is more painful in the last days.

There were a few differential diagnoses like Myasthenia gravis, including Fishers-disease, Lambert-Eaton syndrome, encephalitis and wound botulism. That’s why we made a CT-scan of the cerebrum and a cerebrospinal fluid examination first. Both of them were without any pathological results. Especially the liquor showed normal high of leukocyte, erythrocyte, protein, lactate and glucose. The examination of the blood serum showed a high CRP (C-reactive-protein) of 10,8 mg/dl but normal leucocytes of 10,8 /nl. All other blood tests were inconspicuous; except the toxic screen for heroin, methadone and paracetamol, which were all positive.

The following CT-scan of the pelvic region showed a typical abscess formation in the subcutaneous and also in the adductor muscular region along with typical inguinal and paraaortal lymphadenopathy. An immediate operation with incision, abscess debridement and insertion of a drainage in general anesthesia was performed.

After the operation we took surveillance on the intensive care unit for one day. The patient showed no further dyspnea, normal blood gas results and no general complications. Complementary we gave a high dose of intravenous Penicillin G (5 M four times a day) for in total nine days. Additionally we gave 2 times trivalent Botulinum antitoxin, which made no complications like allergic reaction or other. The intraoperative taken sample gave the evidence of *Clostridium botulinum* Serotype B in the culture and it also showed up in the patient’s serum. It was sensitive for metronidazole (minimal inhibitory concentration 4 μg/ml) and penicillin (minimal inhibitory concentration 0,06 μg/ml).

In the following days the cranial nerve dysfunction decreased and the patient left the hospital nine days after admission.

A second presentation, 14 days after the patient’s discharge of the hospital, showed a complete regression from the neurological symptoms and no wound healing problems.

## Discussion and conclusions

This case shows a rare, challenging and potential life-saving diagnosis of wound botulism in the ED. There is no test, which can prove the diagnosis in a fast and mostly available way. Patient’s serum or other materials like faeces or vomit can be cultivated in order to determinate *C. botulinum*. Real-time PCR or mouse-assay are two more laboratory diagnostics, which can detect the toxin, but they are often not reachable in a rapid way. Furthermore the existing trivalent antitoxin treatment (A, B, E) is only recommended to be used in the first 24 h because a delayed administration seems to be associated with a poor outcome [7, 8].

Although it is difficult to separate Botulism to other similar diseases like Myasthenia gravis (dilated pupils vs. normal pupils or reflexes absent vs. present) or Guillain-Barré syndrome, which comes with more likely ascending pattern weakness, Botulism has typical symptoms [9]. Because of the effect to the presynaptic ACh-Receptor there are symptoms like ptosis, diplopia, dysphagia, dysphonia, dilated fixed pupils, descending paralysis (early in progress pupillary muscles), respiratory failure and others. If the patient is an intravenous drug user and the clinical examination shows an abscess, the wound botulism seems to be very likely. Fever is one more symptom to differentiate wound botulism to the other forms of botulism [2]. There are no drugs available for post-intoxication treatment [3]. A case from 2015 in Italy presents a wound botulism due to an open bone fracture, detected at least one month after trauma. Because of mild symptoms (ptosis, opthalmoplegia, slurred speech) no antitoxin was given. The patient was treated with operation and antibiotics and recovered without any worsening of symptoms [10].

In conclusion the best treatment for wound botulism is still the rapid surgery debridement, acquirement of several samples and the calculated antibiotic and antitoxic treatment.

The CARE guidelines were followed in this case.

## Abbreviations

ACh-Receptor: Acetylcholine receptors
CRP: C-reactive-protein
CT-scan: Computerized tomography scan
ED: Emergency department
IDU: Intravenous drug users
PCR: Polymerase chain reaction
